# Impact of COVID-19 on older adults and role of long-term care facilities during early stages of epidemic in Italy

**DOI:** 10.1038/s41598-021-91992-9

**Published:** 2021-06-15

**Authors:** Stefano Amore, Emanuela Puppo, Josué Melara, Elisa Terracciano, Susanna Gentili, Giuseppe Liotta

**Affiliations:** 1 Community of Sant’Egidio, Piazza della Nunziata 4, 16124 Genova, Italy; 2 Biomedicine and Prevention Department, University of Rome “Tor Vergata”, Rome, Italy; 3 School of Doctorate in Nursing Science and Public Health, University of Rome “Tor Vergata”, Rome, Italy

**Keywords:** Viral infection, Epidemiology, Geriatrics, Public health

## Abstract

Older adults are the main victims of the novel COVID-19 coronavirus outbreak and elderly in Long Term Care Facilities (LTCFs) are severely hit in terms of mortality. This paper presents a quantitative study of the impact of COVID-19 outbreak in Italy during first stages of the epidemic, focusing on the effects on mortality increase among older adults over 80 and its correlation with LTCFs. The study of growth patterns shows a power-law scaling regime for the first stage of the pandemic with an uneven behaviour among different regions as well as for the overall mortality increase according to the different impact of COVID-19. However, COVID-19 incidence rate does not fully explain the differences of mortality impact in older adults among different regions. We define a quantitative correlation between mortality in older adults and the number of people in LTCFs confirming the tremendous impact of COVID-19 on LTCFs. In addition a correlation between LTCFs and undiagnosed cases as well as effects of health system dysfunction is also observed. Our results confirm that LTCFs did not play a protective role on older adults during the pandemic, but the higher the number of elderly people living in LTCFs the greater the increase of both general and COVID-19 related mortality. We also observed that the handling of the crises in LTCFs hampered an efficient tracing of COVID-19 spread and promoted the increase of deaths not directly attributed to SARS-CoV-2.

## Introduction

In December 2019 in Wuhan (China) a novel coronavirus (SARS-CoV-2) was discovered. The infection spread firstly in Hubei and then in some Chinese regions. Starting from January 2020, cases of COVID-19 infections appeared in several countries around the world^1–5^. Italy was the first country outside China severely affected, starting from the end of February 2020, with the first case of local transmission of COVID-19 reported in Lodi province in Lombardia region on the 21st February,^5–7^ and two clusters of COVID-19 cases in Lombardia and Veneto. Starting from the 24th February Italian National Health Institute (ISS) and Italian Civil Protection daily registered several data linked to COVID-19 outbreak^8–10^.

Despite the containment rules^11^, the trend of daily COVID-19 infections continuously increased for 4 weeks and stabilised around the 22nd of March. Together with the growth of total number of infections the number of deaths attributed to SARS-CoV-2 daily increased up to 969 people dead on the 27th March. At the end of April (29th April) 199,470 incident cases had been detected in Italy by real-time reverse transcriptase PCR (rRT-PCR) testing and 25,215 deaths attributed to SARS-CoV-2^12^. The case fatality rate (CFR), calculated on the official infections and deaths was 7.2% overall and was higher in the elderly and man^12,13^. Onder and co-workers^13^ explain as this CFR was over estimated because patients who presented with less severe clinical disease (and therefore with lower fatality rate) were no tested. However, the number of COVID-19 infections not detected by testing as well as the real number of deaths linked directly or indirectly to the pandemic is debated^14^. It is well known that in all the countries an age dependence as the consequences of COVID-19 infection^15,16^ is observed. In Italy during the first months of pandemic, older adults have been the most affected by COVID-19 severe infection: 25.3% of total infections and 55.3% of deaths involved people aged >80 at the end of April 2020 in Italy^12^. In particular elderly in Long Term Care Facilities (LTCFs) have been dramatically hit by COVID-19 outbreak all around the World^17–25^. LTCFs are highlighted as risk factor in COVID-19 spreading and mortality^26^. Gardner et al.^27^ explains as LTCFs consist of a high-risk population in a high-risk setting, while McMichael and co-workers^28^ describe the epidemiology of COVID-19 in a LTCFs and Etard^29^ presents the impact of worst-case scenarios in French LTCFs using a specific age structure and case-age fatality ratios. Although, several indicators suggest that the same scenario occurred also in Italy, the paucity of reliable data on COVID-19 in LTCFs does not allow a quantitative study on the effects of SARS-CoV-2 into these structures. ISS published a Survey on the COVID-19 infections in the LTCFs^30^, however few and disaggregated data was collected that involved less than half of the health LTCFs and no Social LTCFs. A very interesting study has been online published (only in Italian) by the health care service (ATS) of Milan^31^. The authors report the high impact of COVID-19 on the mortality in LTCFs due to the characteristics of resident people and velocity of virus diffusion.

ISS and the Italian National Institute of Statistics (ISTAT) produced reports and dataset on the mortality from 1st January 2020^32^. The first report issued was based on data until 31th March. A new dataset including 7357 municipalities was issued on the 9th July^33^ together with a Summary Report^34^. Both reports^32,34^ showed a large mortality increase in the regions most affected by COVID-19 outbreak.

In this paper we analyse both COVID-19 Infections data^10^ and mortality increase data^33^ for Italian regions, focusing our analysis on the effects on adults over 80s. The correlation between mortality impact on older adults and LTCFs is explored as well as the role played by LTCFs in COVID-19 tracing and health system saturation.

## Methods

We define a set of indicators to monitor the effects of COVID-19 on the total mortality in older adults and to study the role of LTCFs (in terms of beds and residents in LTCFs at regional level). The correlation between mortality increase in older adults over the last five years and the number of beds/residents in LTCFs is studied. In addition we analyse the growth patterns of COVID-19 cumulative infections and deaths and total mortality increase, during early stage of pandemic in Italy.

**Table 1:** COVID-19 cases incidence and COVID-19 deaths *per* 100,000 in all Italian Regions at 15th April 2020^10^; percentage of regional population covered by municipalities reported in the mortality data-set issued by ISTAT^33^.

Region	COVID-19 cases$^a$	COVID-19 deaths$^a$	Reg. Pop. [%]	> 80 in LTC [%]	LTC beds rate [%]
Valle d’Aosta	763	96.3	91.2	8.7	3.7
Lombardia	615	113.1	98.9	8.1	2.9
Trentino A.A.	507	50.5	93.5	10.8	4.4
Emilia R.	471	62.5	97.3	6.1	3.0
Piemonte	420	46.3	96.0	9.3	4.1
Marche	389	48.9	93.2	5.7	2.2
Liguria	385	52.0	97.5	6.4	2.7
Veneto	298	19.2	92.9	8.0	3.2
Toscana	206	14.9	96.6	3.9	2.0
Friuli V.G.	210	17.4	94.7	8.4	3.2
Abruzzo	174	18.3	93.2	3.1	1.3
Umbria	150	6.6	95.4	3.0	1.3
Lazio	89	5.3	93.4	2.7	1.3
Molise	87	4.9	94.7	3.4	2.0
Puglia	80	7.1	95.5	2.5	1.2
*Sardegna*	71				
*Campania*	66				
*Basilicata*	57				
*Sicilia*	51				
*Calabria*	50				

$$^a$$ *per* 100,000

Last two columns report the percentage of over 80 in LTCFs^35^, and the LTCFs beds rate^36^. Only Regions with more the 80 cases *per 100,000* are considered. (Trentino A.A.: Trentino Alto Adige; Emilia R.: Emiglia Romagna; Friuli V.G.: Friuli Venezia Giulia.)

Table 1 reports cumulative infections *per 100,000* in each Italian region at 15th April 2020^10^; the population of the municipalities considered in mortality dataset issued by ISTAT^33^; corresponding percentage compared to the total regional population; number of beds in LTCFs^36^ and number of over 80 in LTC^35^. These last two sets of data refer to 2016 which are the last official data available. The impact of COVID-19 outbreak in the Italian Regions has been very uneven. Lombardia and other regions in North of Italy have a very high infection rate, ranging between 763 case *per 100,000* in Valle d’Aosta to 389 in Veneto. Lower values have been observed in the centre regions such as Toscana (206 *per 100,000*) and even less in South of Italy. These data highlight a strong unevenness in COVID-19 diffusion among Italian regions. For this reason it is necessary to avoid misleading interpretation of the consequences of the pandemic (e.g. COVID-19 mortality, increase of mortality etc) due to the strong differences in the infections rates. According to this, we have applied an arbitrary cutoff at 80 cases *per 100,000* for excluding the regions where the impact of COVID-19 was not relevant. It is worth to underline as the regions excluded from the analysis present low values for over 80s in LTCF and LTCF bed’s rate. Therefore, our approach allows to reduce the effects of the different COVID-19 diffusion among the regions on the parameters we take into consideration for this study, avoiding the overestimation of the correlation between the increase of mortality and the LTCFs.

In this paper two datasets are used:

- the time evolution of the cumulative number of COVID-19 cases and deaths in Italian Regions in the period between 24th February and 15th April 2020, as reported by official daily bulletin of Italian Civil Protection^10^. In this period the reported infections consist only of symptomatic patients, positive at the COVID-19 PCR. In the early stage of pandemic, due to the emergency conditions, the number of PCR tests was quite limited (from about 4334 PCR tests on 21st February to 56,000 test on 11 April). The data are available on the public repository of the Italian Government and are daily collected by each Italian region and cover 100% of Italian municipalities and populations.
- the data on daily mortality (for all causes, not only connected to COVID-19) published by ISS-ISTAT for 7357 Italian municipalities (93.1% municipalities) for the period between 1st January to 31st May 2020 and the corresponding period of last five years 2015–2019^33^. The population coverage ranges, between 100 and 68%, at provincial level; between 98.8 and 85% at regional level; resulting in the average value of 95% for Italian total population. In the dataset, 547 Italian municipalities (7904 total number of Italian municipalities) have been excluded due to not reliability of the collected data. The high coverage of the entire population considers these data representative of the entire population concerning both gender and age distribution. Because, in Italy, at the time of this study, the highest impact of pandemic has been observed until the first half of April and the data up to 15th April are more consolidated, only the period 1st January–15th April is analysed in this paper.

As general rule in all the tests and statistical measures used in the following analysis, the significance threshold was set at 0.05.

### Mortality increase, mortality rate and excess of deaths

The dataset of mortality in Italy^33^ includes the daily number of deaths by sex and age class. For the daily mortality analysis the daily figures are first summed up by region; for each age class we calculate the average value between 2015 and 2019 and then the percentage increment ($\Delta [\%]$) as a function of time.

Cumulative deaths increase in the period 1st March–15th April, is also calculated for over 65, and over 80 (Table 2) with the corresponding ninety five percent confidence intervals (95% CI) according to Altman et al.^37^.

In order to ensure the statistical significance of the comparison between the mean values 2015–2019 and 2020 we firstly verified, by graphical analysis and KPSS test^38^, the stationarity of the time series for the deaths *per* 100,000 among over 65 and over 80. Then, we applied the one-sample t-test^39^ for normal distributed samples, or Wilcoxon Signed Rank Test^40^ for not-normal distributed samples, for each region, between the time series 2015–2019 and 2020. The normality of data has been checked by Ryan-Joiner test (Shapiro-Wiki like). We observed that the stationarity is verified for all time series. In addition the statistical significance of the difference between the mean value 2015–2109 and 2020 is not verified only for Molise, Lazio and Umbria for both over 80 and over 65, according with the low values (even negative for Lazio) of the percentage deaths increases as reported in Table 2. More details regarding the stationarity analysis, one sample t-test and Wilcoxon test are provided in “Supplementary Materials”.

Aiming to estimate the discrepancy between the increase of mortality and the number of deaths officially attributed to COVID-19 we introduce a parameter named: Excess of deaths in the general population (E *per 100,000*). E is the difference of increase of deaths in 2020 and deaths with COVID-19 diagnosis^41^. Because the data of increase of deaths reported in ISTAT dataset^33^ do not consider the entire Italian population we multiplied the increase of deaths in the study municipalities by the corresponding 2020 population, obtaining the estimated increase (this estimation is justified by the high coverage of the population reported in the ISTAT dataset^33^) of deaths in the entire population ($\Delta _d$):

1 $$\begin{aligned} \Delta _d=\left[ n_d(2020)-n_d(15-19)\right] \cdot \frac{100{,}000}{N_m} \end{aligned}$$

with $n_d(2020)$ and $n_d(2015-19)$ the number of deaths in 2020 and the average value 2015–19 in the studied municipalities, and $N_m$ the number of inhabitants of the studied municipalities. Then, we calculate the number of deaths attributed to COVID-19 for each region *per 100,000*:

2 $$\begin{aligned} D_{C19}=d_{C19}\cdot \frac{100{,}000}{N_{tot}} \end{aligned}$$

where $d_{C19}$ is the number of death attributed to COVID-19 and $N_{tot}$ is the total population for each region.

Finally the Excess of Deaths is defined:

3 $$\begin{aligned} E[per \,\, 100{,}000]= \Delta _d-D_{C19} \end{aligned}$$

The cumulative values of E in the period 1st March to 15th April are reported in Table 2.

**Table 2:** Percentage deaths increase in the period 1st March to 15th April 2020 vs 2015–2019 for 7357 municipalities: $\Delta _{>80} [\%]$ for over 80s; $\Delta _{>65} [\%]$ for over 65 s.

Region	$\Delta _{>80} [\%]$	95% CI	$\Delta _{>65} [\%]$	95% CI	E [*per 100,000*]	95% CI
Valle d’Aosta*	100.2	(70.5–142.4)	90.5	(60.6–136.9)	31.4	(17.5–45.4)
Lombardia*	186.8	(184.5–189.1)	189.2	(186.7–191.6)	116.5	(116.0–117.1)
Trentino A.A.*	88.10	(76.5–101.5)	85.3	(72.7–100.8)	44.1	(39.9–48.3)
Emilia R.*	68.7	(66.0–71.4)	73.0	(70.0–76.4)	44.1	(42.8–45.4)
Piemonte*	73.2	(70.1–76.5)	68.8	(65.4–72.6)	57.3	(55.7–58.9)
Marche*	50.9	(44.1–58.4)	53.4	(45.1–58.4)	27.9	(24.1–31.8)
Liguria*	68.7	(61.8–76.2)	69.5	(62.0–78.5)	68.0	(63.7–72.4)
Veneto*	36.7	(34.4–39.2)	31.7	(29.3–34.5)	19.5	(18.3–20.7)
Toscana*	20.7	(18.2–23.3)	19.0	(16.4–22.1)	13.5	(11.2–15.2)
Friuli V.G.*	24.9	(18.4–32.2)	19.4	(12.8–27.4)	10.8	(11.7–15.3)
Abruzzo*	17.4	(11.5–23.9)	15.4	(9.13–23.1)	6.8	(2.5–11.1)
Umbria	10.3	(4.0–17.5)	6.9	(0.2–15.2)	3.6	(− 1.6–8.9)
Lazio	1.1	(− 1.3–3.6)	− 0.68	(− 3.2–2.2)	− 7.4	(− 8.6–6.3)
Molise	13.3	(2.6–26.3)	5.5	(− 5.2–20.2)	1.3	(− 8.1–10.8)
Puglia*	15.8	(13.2–18.5)	13.4	(10.7–16.5)	10.3	(8.7–11.8)

Corresponding 95% CI is reported. Value of E are reported *per 100,000* with the corresponding 95% CI. Complete data analysis regarding stationarity and statistical significance can be read in the section “Supplementary Materials”.

*Statistical significance for t-test/Wilcoxon Signed Rank Test.

Starting from mortality and demographic data^41^, Mortality Rate in the period 1st March to 15th April is calculated for class age over 80 years (MR$_{>80}$) dividing the number of deaths by the corresponding population, for both 2020 and 2015–2019. Because the coverage of population is, for all regions, greater than 90% we assumed the mortality rates of the municipalities of study representative of the entire regional population without any standardisation as done by Magnani *et al.*^42^. Finally, the Rate Ratio (RR$_{>80}$) is computed dividing the Mortality Rate 2020 (MR$_{>80}$(2020)) by Mortality Rate (2015–2019) (MR$_{>80}$(2015–2019)) (Table 3).

**Table 3:** Mortality rate *per 100,000* in over 80, by region in last five years (MR$_{>80}$(2015–19); in 2020 (MR$_{>80}$(2020); and Rate Ratio (RR) and 95% CI for the period 1st March to 15th April.

Region	MR$_{>80}$(2015–19) [*per 100,000*]	95% CI	MR$_{>80}$(2020) [*per 100,000*]	RR	95% CI
Valle d’Aosta	1314.3	(1279.2–1351.5)	2529.7	1.92	(1.98–1.87)
Lombadia	1216.5	(1165.8–1271.8)	3266.7	2.69	(2.80–2.57)
Trentino A.A.	1256.0	(1216.7–1297.9)	2255.7	1.80	(1.85–1.74)
Emilia R.	1292.8	(1264.7–1322.1)	2103.5	1.63	(1.66–1.59)
Piemonte	1285.0	(1244.1–1328.8)	2119.9	1.65	(1.70–1.60)
Marche	1250.4	(1222.9–1279.2)	1821.0	1.46	(1.49–1.42)
Liguria	1291.0	(1266.1–1316.9)	2112.8	1.64	(1.67–1.60)
Veneto	1267.2	(1224.3–1313.2)	1647.4	1.30	(1.35–1.25)
Toscana	1300.8	(1269.8–1333.3)	1510.4	1.16	(1.19–1.13)
Friuli V.G.	1308.1	(1269.0–1349.7)	1558.8	1.19	(1.23–1.15)
Abruzzo	1315.5	(1292.6–1339.2)	1509.2	1.15	(1.17–1.13)
Umbria	1305.4	(1279.5–1332.3)	1391.3	1.07	(1.09–1.04)
Lazio	941.1	(928.1–954.5)	955.4	1.02	(1.03–1.00)
Molise	1275.5	(1228.1–1326.8)	1221.6	0.96	(0.99–0.92)
Puglia	1301.1	(1258.8–1346.3)	1436.9	1.10	(1.14–1.07)

### Power-law scaling

The analysis of growth patterns from the beginning of the pandemic suggested COVID-19 to spread exponentially^43,44^, which is consistent with other epidemics and epidemiological theories^45^. However, a comprehensive analysis, among several countries, of the growth dynamics of this infection in the early stages of diffusion highlights that while some countries can be better described by exponential growth, many other countries are more accurately described by a power-law^46^ consistently with what observed at the beginning of pandemic in China^47^ and in other countries^48,49^. In particular Komarova and co-workers^46^ observed that Italy and other European countries show a power-law like growth during the first period of diffusion. In our study, we analysed the growth patterns of cumulative number of infections, the number of COVID-19 attributed deaths and the cumulative increase of total deaths for each region in the first 30 days starting from first reported case. The spreading kinetics of cumulative cases, within the aforementioned time shift, does not show any saturation. For this reason logistic law, as well as Richard’s or modified Richard’s law are not taken under consideration, while could be suitable in further stages of pandemic. We fitted cumulative infections of each region with both power-law and exponential. Fitting results show that for all Italian regions spreading kinetics follows a power-law like growth as reported by Komarova for Italy^46^ (the details of comparison between power-law and exponential are provided in the “Supplementary Materials”).

In this work Eq. (4) is used to fit the number of COVID-19 infections, the number of COVID-19 attributed deaths and the cumulative increase of total deaths for each region.

4 $$\begin{aligned} N(t)=B\cdot t^\alpha \end{aligned}$$

**Figure 1:**
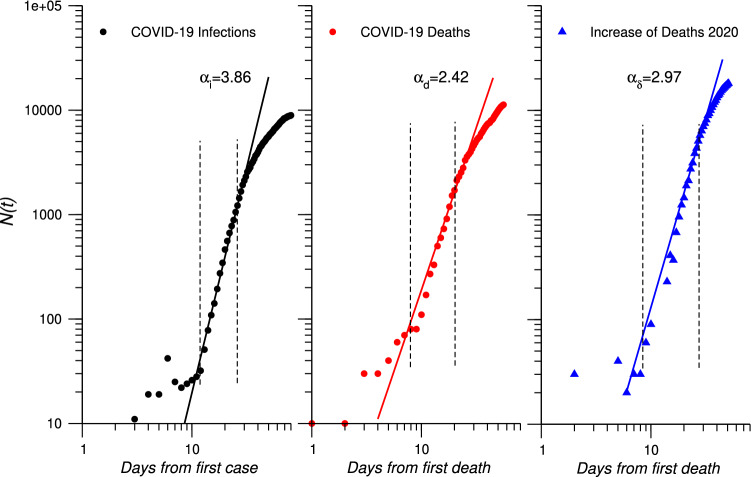
Log–Log plots of total cumulative number of COVID-19 infections starting from 25th February (first case in Liguria), cumulative number of COVID-19 deaths and cumulative increase of deaths starting from 4th March (first deaths in Liguria) for Liguria until 15th April 2020. Best fit lines according to Eq. (4) are superimposed to the observed data. Vertical dashed lines indicate the fitting range

We report data in a log-log plot starting from: the day of the first case for the number of infections and the day of the first deaths attributed to SARS-CoV-2 for number of deaths and increase of total deaths (Fig. 1 for Liguria). The range used to extract the scaling exponents is denoted by two vertical dashed lines. The best fit line is superimposed on the the data points. The three scaling exponents for cumulative infections ($\alpha _i$), cumulative COVID-19 deaths ($\alpha _d$) and cumulative increase of deaths ($\alpha _\delta$) are extracted and reported in Table 4.

**Table 4:** Extracted scaling exponents by region, for cumulative infections ($\alpha _i$); cumulative COVID-19 deaths ($\alpha _d$); cumulative increase of deaths in 2020 ($\alpha _\delta$) and difference between $\alpha _\delta$ and $\alpha _d$.

Region	$\alpha _i$	$\alpha _d$	$\alpha _\delta$	$\alpha _\delta -\alpha _d$
Valle d’Aosta	3.29	2.38	2.33	0.05
Lombardia	3.51	3.04	2.43	0.61
Trentino A.A.	2.78	1.88	1.91	0.08
Emilia R.	2.91	2.90	3.00	0.10
Piemonte	3.37	2.28	2.38	0.10
Marche	2.86	2.90	2.94	0.04
Liguria	3.86	2.60	2.97	0.37
Veneto	3.28	2.28	1.62	− 0.66
Toscana	3.12	1.76	1.71	− 0.05
Friuli V.G.	2.49	1.72	1.93	0.21
Abruzzo	3.40	2.78	2.25	− 0.53
Umbria	3.09	1.16	0.02	− 0.96
Lazio	3.24	2.12	0.00	− 2.12
Molise	2.38	0.8	0.00	− 0.80
Puglia	3.18	2.32	1.33	− 0.99

The scaling exponent $\alpha _i$ indicates the velocity of growth of the infections, while $\alpha _d$ and $\alpha _\delta$ the velocities of growth of deaths attributed to COVID-19 and of increase of total deaths, respectively. The difference $\alpha _\delta -\alpha _d$ (reported in Table 4) monitors the mismatch between the time evolution of increase of total deaths and the growth of the number of COVID-19 deaths.

### Correlation analysis

In order to quantitatively study the role played by LTCFs during COVID-19 infection, the relation between the introduced parameters, related to the increase of mortality, and two independent variables linked to LTCFs–LTCFs beds rate and percentage of over 80 in LTCF (see Table 1)—is explored. For this purpose two set of indicators can be distinguished:

- $\Delta _{>65}$; $\Delta _{>80}$; RR$_{>80}$: to explore the correlation between LTCFs and the mortality increase in older adults;
- E; $\alpha _{\delta }-\alpha _e$ to explore the correlation between LTCFs and the ability to follow the COVID-19 outbreak as well as the effects of health services disruption.

We aim to define the degree of correlation between independent and dependent variables by using parametric (e.g. Pearson) or not parametric (e.g. Spearman) coefficients. The use of Pearson’s correlation coefficient requires that data are bi-normal distributed, while for Spearman’s rank correlation coefficient normality is not required^50,51^. By using Ryan-Joiner test the normality of variable’s dataset is verified only for the two independent variables (LTCFs beds rate and percentage of over 80 in LTCFs) while all the dependent variables are not normally distributed (details on the normality check are provided in the “Supplementary Materials”). For this reason we applied the Spearman correlation coefficient to test the correlation strength between independent and dependent variables using the significance threshold 0.05 for the *p*-value and the ranges as reported in Table 5 for the strength of correlations:

**Table 5:** Interpretation of Spearman’s correlation coefficients as used in the work

Range of corr. coeff.	Correlation strength
1 (-1)	Perfect
0.8 (− 0.8)–0.9 (− 0.9)	Very strong
0.6 (− 0.6)–0.7 (− 0.7)	Moderate
0.3 (− 0.3)–0.5 (− 0.5)	Fair
0.1 (− 0.1)–0.2 (− 0.2)	Poor
0	Null

In addition, multivariate model approach has been applied to explore the correlation between mortality increase in over 80 and independent variables. A step-wise approach has been followed starting from one single variable adding and removing others independent variables. The goodness of the model is controlled by the adjusted-$R^2$ and the statistical significance of the independent variables (*p*-value). We fixed adjusted-$R^2>0.9$ and p-value confidence level 0.05 as reference values. In Table 6, the explored models are summarised:

**Table 6:** Multivariate models explored. Independent variables; *p*-values; and adjusted-$R^2$ are reported for each model.

Model	Variables	p-values	adj-$R^2$
1	Total deaths with COVID	$<0.001$	0.86
2	Total deaths with COVID; % of over 80 infections	$<0.001$; 0.30	0.86
3	Total deaths with COVID; % of over 80 infections; % LTC beds	$<0.001$; 0.98; 0.001	0.95
4	Total deaths with COVID; % LTC beds	$<0.001; <0.001$	0.96

Models 1 and 2 use independent variables only related to the COVID-19 diffusion in each region and among over 80 with a resulting adjusted-$R^2$ lower than 0.9. Then we introduced the % of LTCFs beds rate with an increasing of $R^2$, but the COVID-19 among over 80 jumped out to the model due to the p-value. Model 4 satisfies both $R^2$ and p-value reference values. In addition we checked that the normality of residual is verified only for Model 4.

## Results

### Growth patterns

The analysis of time evolution of COVID-19 infections, COVID-19 deaths and of overall deaths, by power-law approach allows us to define the general behaviour of pandemic kinetics in each region.

Figure 1 shows the log-log plot of total infections, COVID-19 deaths and increase of deaths in Liguria. This region shows the highest mismatch between $\alpha _{\delta }$ and $\alpha _{d}$ excluding Lombardia which presents a particular feature due to the impact of the pandemic. The plots show as power-law fits real data in the period between the two dashed lines corresponding to about 25 to 30 days. An approximately seven days time shift, between infections and deaths (both COVID-19 and increase) is also observed. The scaling exponents for total infections ($\alpha _i$), COVID-19 deaths ($\alpha _d$) and overall increase of deaths ($\alpha _\delta$) are reported in Table 4.

Infection scaling exponent defines three groups of regions: $\alpha _i>3.5$ Lombardia and Liguria; $3.0<\alpha _i<3.5$ Valle d’Aosta, Piemonte, Veneto, Toscana, Abruzzo, Lazio, Puglia, Umbria; $\alpha _i<3.0$ Trentino A.A., Emilia R., Marche, Friuli V.G., Molise. Infections scaling exponent indicates the speed of outbreak spread during the power-law regime. The results show a very fast growth of pandemic in several Italian regions with scaling exponents higher than what observed for example in China (e.g. Hubei region $\alpha _i=2.48$^47^). Although $\alpha _i$ gives indications on the kinetics of the diffusion rate of COVID-19 in each region it does not explain the entire evolution of pandemic because other factors affects its magnitude such as time lapse between first COVID-19 case and lockdown; duration of power-law regime, number of initial cases (registered), total regional population. Despite, Lombardia is the most hit regions, it did not have the highest scaling exponent: infections in Lombardia grew under a power-law regime at least from 24th February to about 25th March before the attenuation due to the lockdown which was very efficient to slow down the epidemic diffusion as showed by Sebastiani^52^. On the contrary, in region such as Abruzzo the power-law regime, with a quite high scaling exponent, was observed for a very limited time range because it started closer to the attenuation effects of restriction rules. However, all regions show $\alpha _i>2$ thus greater than a quadratic time evolution.

Scaling exponent $\alpha _d$ indicates the growth rate of COVID-19 deaths. In this case four groups of regions can be identified: $\alpha _d>3.0$ Lombardia; $2.5<\alpha _d<3.0$ Emilia R, Marche, Abruzzo, Liguria; $2.0<\alpha _d<2.5$ Valle d’Aosta, Piemonete, Veneto, Lazio, Puglia; $1.5<\alpha _d<2.0$ Trentino A.A., Toscana, Friuli V.G.; and $\alpha _d<1.5$ Umbria and Marche. The values of $\alpha _d$ are lower than $\alpha _i$ for each region as also reported by Li^47^ for China. As expected Lombardia shows the highest scaling exponents because the highest mortality observed in that region^53^. However, several other regions have scaling exponent higher than 2, meaning a fast growth of COVID-19 deaths. Finally, four groups can be defined by $\alpha _\delta$: $\alpha _{\delta }>2.5$ Emilia R., Liguria and Marche; $2.0<\alpha _{\delta }<2.5$ Valle d’Aosta, Lombardia, Piemonte, Abruzzo; $1.5<\alpha _{\delta }<2.0$ Trentino A.A., Veneto, Toscana, $\alpha _{\delta }<1.5$ Umbria, Lazion, Molise, Puglia. The difference between the two scaling components related to deaths ($\alpha _d$, $\alpha _\delta$) allows to monitor the mismatch in the time evolution of COVID-19 deaths and increase of deaths in 2020. The results show both positive and negative values. Negative values indicate that COVID-19 deaths grew faster than the increase of overall deaths. In contrast, positive values reflect faster growth of overall increase of deaths than COVID-19 deaths. All regions less impacted by epidemic show negative values of the difference $\alpha _{\delta }-\alpha _d$, while regions with similar infection rates such as Marche, Liguria, and Veneto have strongly different values of the difference. Lombardia and Liguria, as aforementioned, have the highest values, showing the big mismatch between increase of deaths and COVID-19 deaths. This last results arise from both undiagnosed and the effects of health system dysfunction.

### Mortality increase

Table 2 reports the percentage increase of deaths in over 80s ($\Delta _{>80}$) and over 65s ($\Delta _{>65}$). Both values show a strong unevenness among regions ranging between +189.2% for over 65 in Lombardia to $-0.68$% in Lazio as showed also by Michelozzi^54^ in the analysis of rapid mortality surveillance system for the major Italian cities. Lombardia shows values widely larger than other regions. Higher values for both $\Delta _{>80}$ and $\Delta _{>65}$ have been observed for regions with higher COVID-19 incidence rates, as expected. However, there are some discrepancies: Liguria has a lower incidence rate than Marche but higher mortality increase and the same is for Piemonte compared with Emilia R in over 80. Rate Ratio in over 80 (RR$_{>80}$) shows values greater than one for all regions excluding Molise. This means that mortality in over 80 in 2020 is higher than in the last five years in the reference period according to what observed by Magnani and co-workers for over 60^42^. Highest value is observed in Lombardia where MR$_{>80}$(2020) is more than 2.5 times than MR$_{>80}$(2015–19). Despite RR$_{>80}$ is strongly influenced by COVID-19 diffusion is not perfectly scaled by COVID-19 incidence rate, similarly to what observed for $\Delta [\%]$.

**Figure 2:**
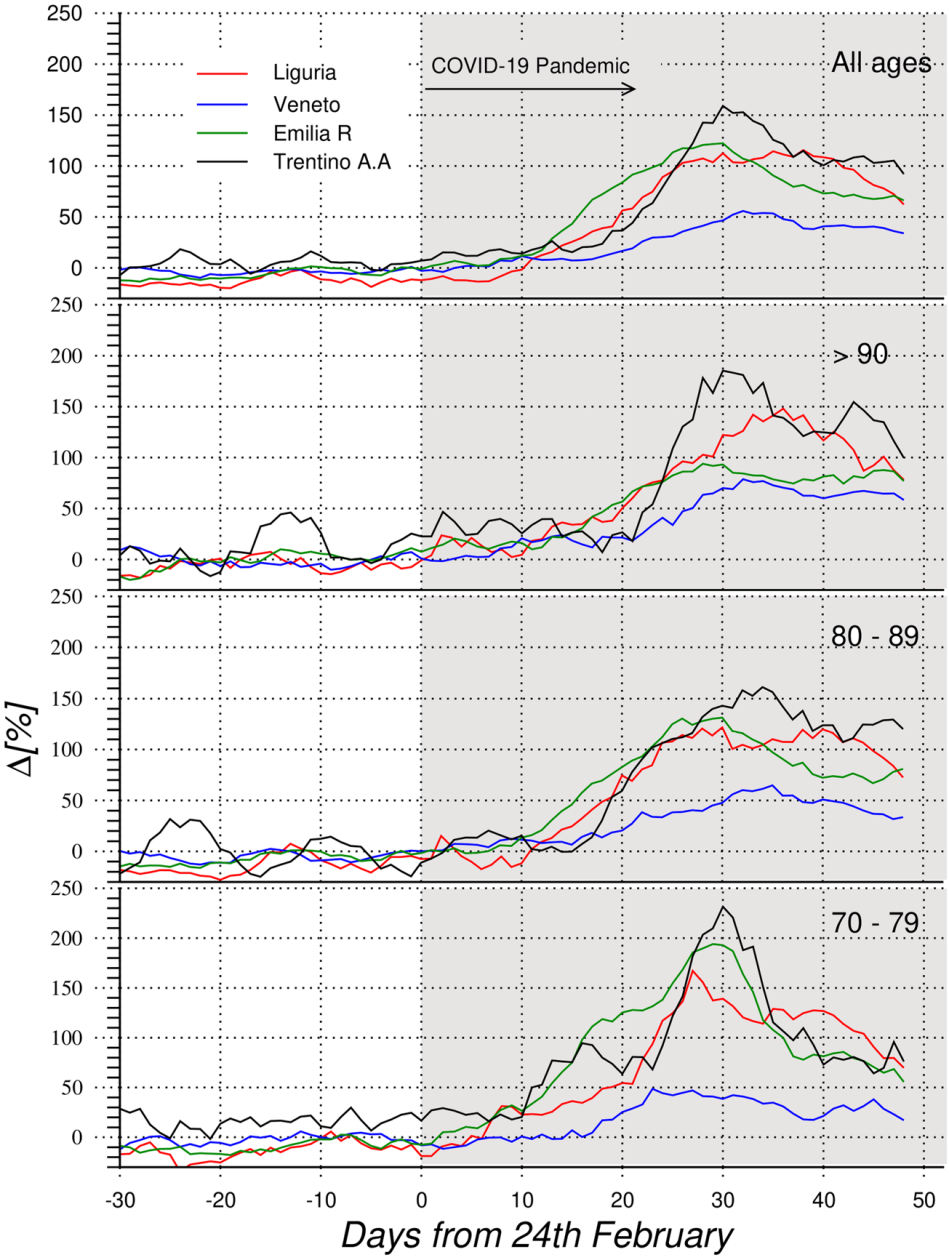
Percentage increase $\Delta [\%]$ of daily mortality in Liguria (red line), Veneto (blue line), Emilia R. (green line) and Trentino A.A. (black line), for age classes. Starting from bottom: 70–79 years; 80–89 years; older than 90 years; all resident people. Seven days running average is applied.

The exploration of time evolution of increase of deaths allows a better understanding of the phenomena. The daily trend of increase of deceases in four Italian regions, Liguria, Veneto, Emilia R., Trentino A.A. is reported in Fig. 2 for 3 age classes and overall. Seven days running average is used to reduce the noise in the plots.

These four regions are representative of some typical trends: high impact for Trentino A.A., Liguria and Emila R., and low impact for Veneto. Other regions, such as Lazio, have a very low impact as reported^34^. The data of Lombardia are not reported in the plot because, of the tremendous impact of COVID-19 in that region, are such high to be not significant compared with other regions.

Starting from the end of February all trends increase, showing a peak of different magnitude, depending on age class and region. While Veneto shows a smooth trend in all the plots, other three regions have more pronounced peaks. Trentino A. A. and Emilia R. present the highest increase of deaths for age classes 70–79, around +200%, while Liguria shows similar values (around + 150%) for all age classes. In addition the peak of 70–79 is sharp for Trentino A.A. and Emilia R., while more spread for Liguria. On the other hand, Trentino A.A., Emilia R. and Liguria show broadened peaks for age classes 80–89 and $>90$. The time evolution is quite similar for all the regions reported in Fig. 2: increase of deceases starts firstly for 70–79, while for 80–89 and in particular for 90+ a delay of approximately 7 to 10 days is observed. The behaviour of 80-89 and 90+ trends shows broadened peaks or double peaks as in Liguria and Trentino A.A. This evolution suggests that COVID-19 started hitting firstly older adults with higher level of mobility and with social contacts (age class 70–79) then people with lower mobility and isolated in LTCFs. It is interesting to observe that the step rise is sharper for regions with a higher number of over 80 in LTCFs such as Trentino A.A. This analyses suggests that as soon COVID-19 reached LTCFs it spread with high speed and with a strong impact on the mortality, with a similar effect to what observed in other countries^55^.

**Figure 3:**
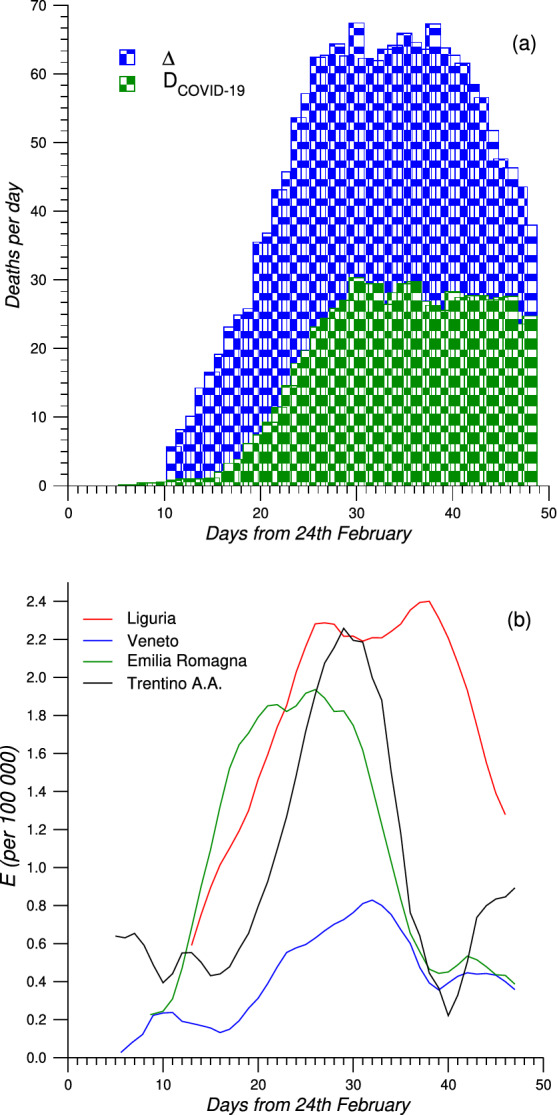
(**a**) Daily deaths attributed to COVID-19 (D$_{COVID-19}$) and daily increase of deaths ($\Delta$) for 2020 vs 2015–2019 in Liguria between 24th February and 15th April. (**b**) E indicator for Liguria (red line), Veneto (blue line), Emilia R. (green line) and Trentino A. A. (black line).

Figure 3a reports the daily deaths attributed to COVID-19 and the daily increase of deaths in 2020 for Liguria. Figure 3b shows *E* for four Italian regions: Liguria; Veneto; Emilia R.; Trentino A.A., as a function of time, starting from the first deaths attributed to COVID-19 for each region.

The mismatch between COVID-19 deaths and increase of deaths in 2020 is clear in Fig. 3a for Liguria. Between the end of February and beginning of March deaths started to increase while the number of COVID-19 deaths is still close to zero. At this stage the excess of deaths observed can be attributed to Sars-CoV-2 infections without diagnosis because at the beginning of the pandemic the health system had not yet collapsed. However, the discrepancy between COVID-19 deaths and increase of deaths continuously grows until the end of March (30 days after 24 February). This trend is more appreciable by the plot of E reported in Fig. 3b. Liguria in particular shows a double peak with the second maximum located in the same date range of the peak of $\Delta _{80-89}[\%]$ and $\Delta _{90+}[\%]$. A similar behaviour is observed also for the others regions even if less pronounced. This means that the peaks in the increase of deaths corresponds to a maximum in unidentified cases or in indirect deaths due to the collapse of health services. The trend of E confirms what inferred by the scaling exponents concerning the growth rates. Despite cumulative values of E for each region, reported in Table 4, obviously are influenced by the COVID-19 diffusion, are not perfectly scaled by the incidence rate as well as observed for $\Delta [\%]$ and RR$_{>80}$. Thus, Liguria has a higher value of E than all other regions, excluding Lombardia, even if it has not the highest incidence rate.

This behaviour is the same observed exploring the discrepancy of the scaling exponents. The combination of these results highlights the hard difficulties of Liguria to both identify COVID-19 cases and deaths and in the collapse of the health system. This last results suggest to explore which other factors, in addition to COVID-19 diffusion, affected mortality among older adults and the mismatch with the COVID-19 deaths.

### Univariate and multivariate analyses

Table 7 reports the results of univariate (Spearman’s correlation coefficients) between the dependent variables and the two independent variables. All values of Spearman’s coefficient ($\rho _s$) in Table 7 are calculated excluding Lombardia. The tremendous impact of COVID-19 on that region suggests not using its data in the correlation study. All Spearman coefficients are greater than 0.7 meaning very strong (0.8–0.9) or moderate correlation (0.7) between variables. In particular all correlations between mortality increase in over 80 and over 65 variables and LTCFs variables are very strong. This is the quantitative confirmation of general statement of the particular burden of LTCFs during COVID-19 epidemic. The mortality among older adults was definitely higher in the regions where a higher number of over 80 is in LTCFs.

**Figure 4:**
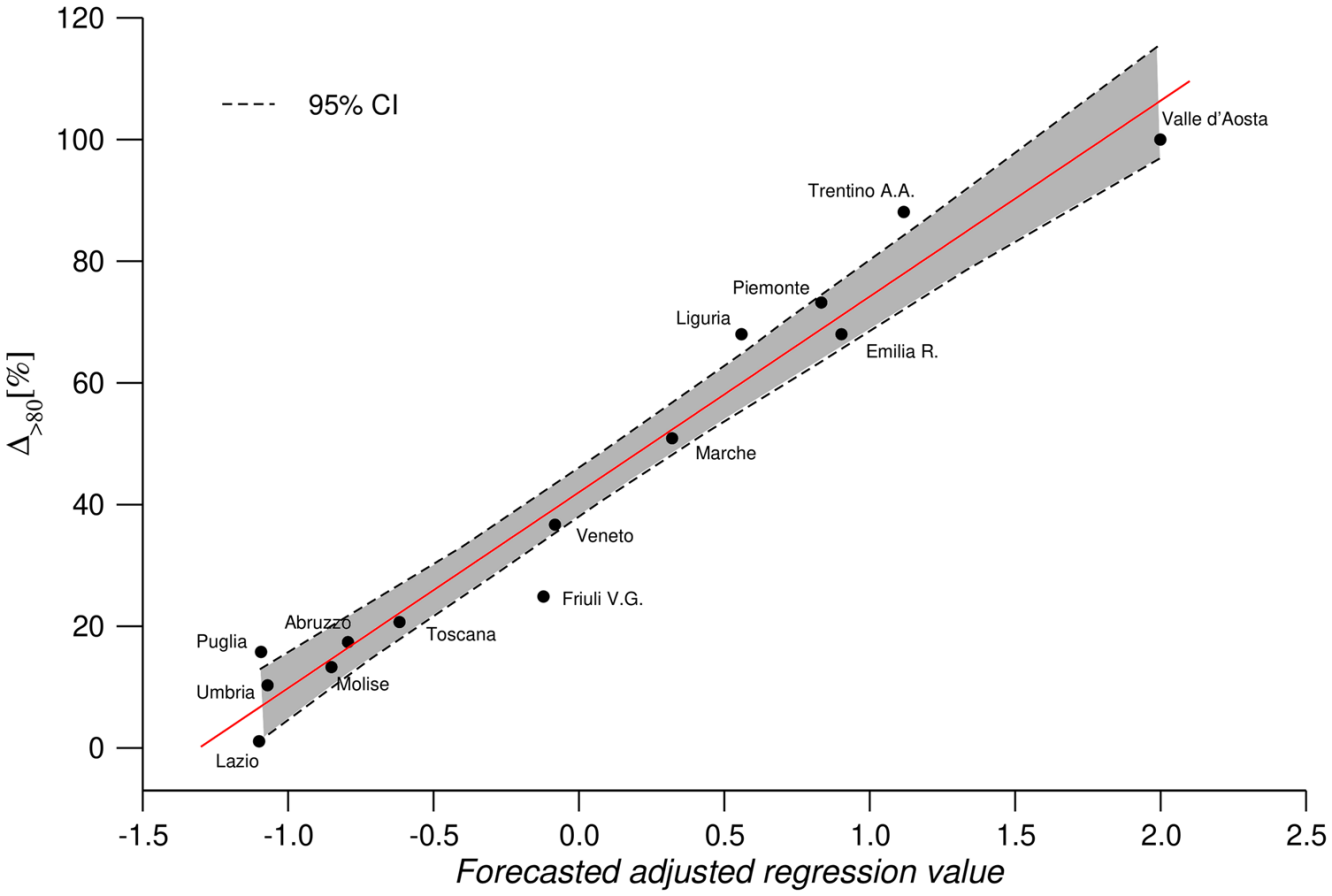
Deaths increase in aged $>80$ population, adjusted for the number of death attributed to COVID-19 and for the LTCFs beds rate (adjusted $R^2$: 0.952, Stat. Sig. <0.001).

This result is confirmed by the multivariate model reported in Fig. 4. As mentioned, we applied a step-wise model starting from the use of independent variables only related to impact of COVID-19 in each region and on over 80: regional incidence rate, COVID-19 incidence in over 80, regional COVID-19 deaths (see Table 1). The results of these models showed adjusted-$R^2$ lower than 0.90 and variables with no statistical significance as reported in Table 6. The final model presented in Fig. 4 uses the mortality increase of over 80 as dependent variable and the LTCFs beds rate and the number of COVID-19 deaths (*per 100,000*) as independent variables. The linear relation observed in Fig. 4 has a adjusted-$R^2$ equal to 0.962 and both variables show significance values lower than $5 \times 10^{-3}$. Despite, it is clear that COVID-19 incidence rate plays a crucial role on the mortality increase our model, taking into account the LTCFs influence, provides a better description of the behaviour of different regions. Model explains why Liguria has higher mortality increase of Marche even if the two regions have similar incidence rates and similarly for Piemonte and Emilia R.

Univariate analysis is also applied to explore the dependence of E and $\alpha _{\delta }-\alpha _e$ on the LTCFs variables. Tables 7 reports value higher than 0.7 for all four $\rho _s$ coefficients, meaning a moderate correlation between dependent variables and LTCFs variables. This result suggests that the higher is the number of older adults in LTCFs the higher is the discrepancy between deaths increase and deaths attributed to COVID-19. This mismatch can arise from two factors: a significant number of deaths in LTCFs due to Sars-CoV-2 have been not registered because not tested with PRC, and the number of deaths for other reasons increased due to the saturation of health service and assistance in LTCFs.

**Table 7:** Spearman’s correlations ($\rho _s$-coefficients) among dependent variables deaths increase in aged >65 ($\Delta _{>65}$ [%]) and aged >80 ($\Delta _{>80}$ [%]), Mortality Ratio Rate (RR), Excess of deaths (E), scaling exponents difference ($\alpha _{\delta }-\alpha _e$) and LTCFs independent variables are reported.

		$\Delta _{>80}$ [%]	$\Delta _{>65}$ [%]	**RR**$_{>80}$	**E**	$\alpha _\delta -\alpha _d$
$>80$ in LTC [%]	$\rho_s$	**0.880**	**0.829**	**0.873**	**0.754**	**0.774**
	*p-value*	<0.001	<0.001	<0.001	*0.002*	*0.001*
LTC beds rate [%]	$\rho_s$	**0.864**	**0.814**	**0.850**	**0.726**	**0.722**
	*p-value*	<*0.001*	<*0.001*	<*0.001*	*0.003*	*0.004*

Bold value indicate the relevant results.

## Discussion

The impact of COVID-19 outbreak in Italy in the period between end of February up to 15th April 2020 is explored focusing on the effects on mortality increase among older adults and the correlation with Long Term Care Facilities (LTCFs). The analysis of several indicators and dataset allows to have a comprehensive understanding of all phenomena and to overcome the paucity of reliable official data on the impact of COVID-19 in Italian LTCFs.

Our study starts from the analysis of COVID-19 growth patterns showing a power-law scaling for all regions although with very uneven magnitude. The increase of deaths among older adults aged >80 ($\Delta _{>80}$[%]) and aged >65 ($\Delta _{>65}$[%]) as well as in mortality rate MR$_{>80}$ are not homogeneous among different regions reflecting different impact of COVID-19 outbreak. However, the results clearly show that COVID-19 incidence rate does not fully explain the differences of mortality impact in older adults among different regions. A correlation between mortality in older adults and number of people in LTCFs is quantitatively observed confirming the tremendous impact of COVID-19 on LTCFs. In addition, correlation between LTCFs and undiagnosed cases as well as effects of health system dysfunction is also demonstrated, showing the limits of LTCFs during the epidemic.

The reasons for the inadequacy of LTCFs to face the spread of the infection and its deadly consequences on the hosts as well as the effects of social isolation and loneliness due to COVID-19 control measure^56^ are under debate worldwide. The explanation related to the severity of hosts’ medical condition^30,31^ are probably one of the factor but not the only one. The unpreparedness of LTCFs to deal with an epidemic is also a contributing factor, because these structure are mainly thought for a daily care of not/partially self-sufficient individuals who need a considerable amount of personal care, several times per day. In this case the precautions to prevent the spreading of an infectious diseases, which is asymptomatic in most of the cases, need a very high level of specific training for the staff and a good staff/host ratio to avoid the staff rotation across different wards and, within the same ward, across different hosts. All these conditions are not usual in the LTCFs^57^. However, the debate is also whether we are observing a specific problem due to a pandemic that are challenging the older adults population wherever it is living, or the pandemic simply underlined the inadequacy of a model of care still based on institutional care as a pillar. In some cases the decision of not offering acute hospital care to the LTCFs’ hosts has been taken because of the place where they were living that was thought adequate enough for their needs for care, which was partially contradicted by the very high mortality registered among them. The percentage of COVID-19 deaths attributed to LTCFs’ hosts varies from 25 to 85%^58^: in Italy, although official data are not available some reports estimate the ratio of mortality comparing the LTCFs’ hosts and people aged more than 70 living in the community to be 3:1^59^. Moreover, the general orientation at European level is toward a personal-centred care^60^ that is exactly what cannot be pursued in LTCFs because of the need of rigid protocols to take care of many people who show a very high need for care. The pandemic highlighted to an extraordinary degree something already inherent in the type of LTCFs care: generalised care model are strongly inadequate to an ageing population which needs individualised care plan, involving formal and informal care givers in a friendly and supportive environment that is the more appropriate approach to care for frail population.

This study has some limitations. A general limitation of all the studies on the early stage of pandemic in Italy is due to an initial bias due to the lack of temporal uniformity in the collection of COVID-19 positive swabs and also among different regions in Italy. Infact during first weeks of pandemic contradictory indications regarding the guidelines to test patients have been diffused by Italian institutions. Moreover, there are other limitations strictly regarding this study. First, we analyse the power low scaling exponents as well the Excess of death in the entire population and not only in aged >80 as the COVID-19 deaths per class age are not retrievable from public sources at this stage of the infection. Second, the public data on LTCFs are not update to 2019 but to 2016, however the variation should be not relevant for our results. Finally, all correlations proposed, although statistically robust, cannot figure out an unique causal relationship. Despite these limitations, the wide range of data explored as well as the similar results obtained from different kind of analysis allow us to draw a general figure on the role of LTCFs during early stage of COVID-19 infection in Italy.

Despite not having the smoking gun the large set of evidences allows us to conclude that LTCFs do not play any protective role over older adults during a pandemic even if isolating rules have been applied. On the contrary mortality increase directly or not directly linked to COVID-19 is higher where a higher number of older adults lives in LTCFs. Our results show as forced isolation of elderly is not at all a solution, as could be inferred by the results showed by Dowd et al.^61^, to hamper the COVID-19 diffusion among older adults as already highlighted in a previous work^25^. In addition current results suggest that the handling of the crises in LTCFs hampered an efficient tracing of COVID-19 spread and promoted the increase of deaths not directly attributed to Sars-CoV-2. In particular this last result is in the same direction of what reported by several studies^55,62,63^ highlighting the correlation between LTCFs concentration and size and the acceleration of infection rates and mortality rates in the surrounding communities^55^.

In conclusion our study highlights as also Italian LTCFs have been the focal-point of what has been defined “*The perfect storm*”^64^ of Nursing Home. The results here reported concur to the international discussion regarding the future of public health policies regarding elderly^65^. Our study can support decision-maker, and public health institutions to understand that the current model of LTCFs clearly showed its inadequacy to face with emergency conditions such as a pandemic while home-based options may permit better containment of transmission^20,66^. Rethinking elderly care services might mean that home is the safest place or that a reshape of LTCFs to smaller and more efficient structures is the future of health and social care of elderly.

## Supplementary Information

Supplementary Information.
