# Supplementary material for: Impact of COVID-19 on older adults and role of long-term care facilities during early stages of epidemic in Italy
Source: Sci Rep. 2021 Jun 15;11:12530. doi: 10.1038/s41598-021-91992-9 (PMC8206111; doi:10.1038/s41598-021-91992-9)
Supplement: Supplementary file 1 — Supplementary Information. [file 41598_2021_91992_MOESM1_ESM.pdf]

Impact of COVID-19 on older adults and role of Long-Term Care Facilities during early stages of epidemic in Italy

Stefano Amore[1*], Emanuela Puppo[1], Josuè Melara[1], Elisa Terracciano[2], Susanna Gentili[3], Giuseppe Liotta[2]

[1] *Community of Sant'Egidio, Piazza della Nunziata 4, 16124 Genova, Italy*
[2] *Biomedicine and Prevention Dept., University of Roma "Tor Vergata", Roma Italy*
[3] *School of Doctorate in Nursing Science and Public Health, University of Roma "Tor Vergata", Roma Italy*

** corresponding author: amore.stefano@gmail.com*

Additional Materials

1) Stationarity check and t-test / Wilcoxon test

Starting from the number of deaths among over 80s and over 65s in the studied municipalities we calculated the number of death per 100,000 inhabitants for each region. Then we reported in the following plots the trend for each studied region for years 2015 -2020 for over 80 (for over 65 is similar)

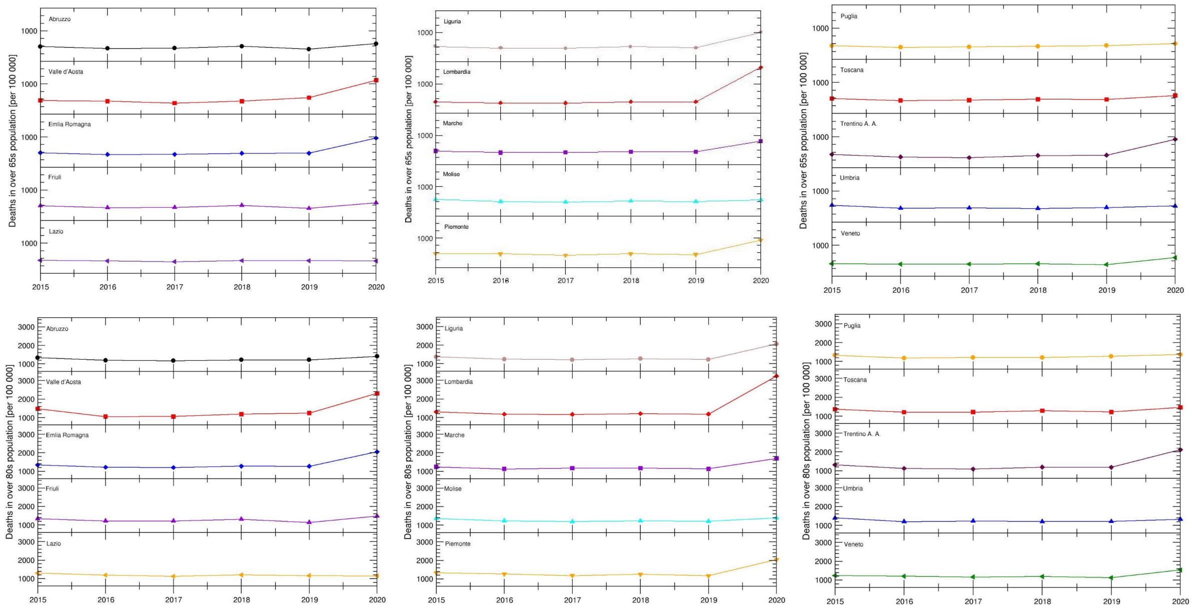

Figure 1: Deaths per 100000 among over 80 and over 65 1st March to 15th April 2015-2020.

These plots clearly show as not trends or anomalies can be observed for in the time series 2015-2019. In addition, we applied the KPSS to test the null hypothesis that a unit root is present in a time series samples 2015-2019. The stationarity of all time series is verified for all regions for both over 65 and over 80 by means of KPSS test. Output of KPSS tests are reported in Appendix 1.

In order to quantify the statistical importance of the impact due to COVID-19 we verified the statistical significance of the difference between the mean values of 2015-2019 and the corresponding single value of 2020. This can be proven by the application of One-Sample t—test for normal distributed samples or Wilcoxon's Rank test for not normal distributed samples. Thus, we firstly checked the normal distribution of data series.

Normally distributed data

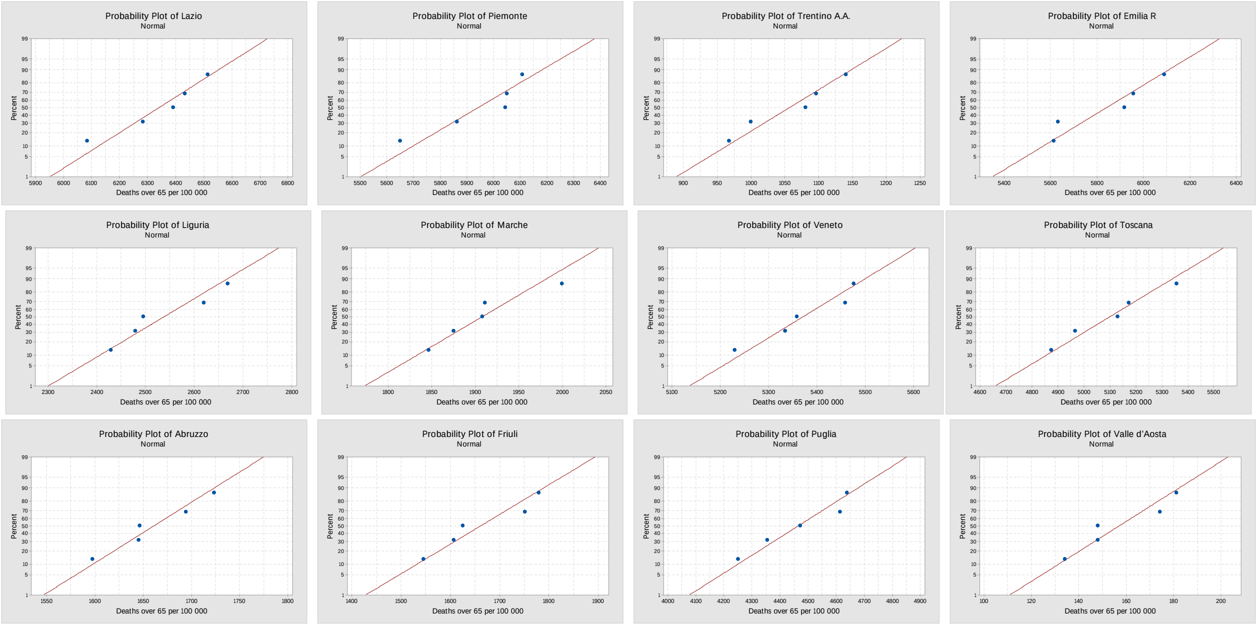

Not-normally distributed data

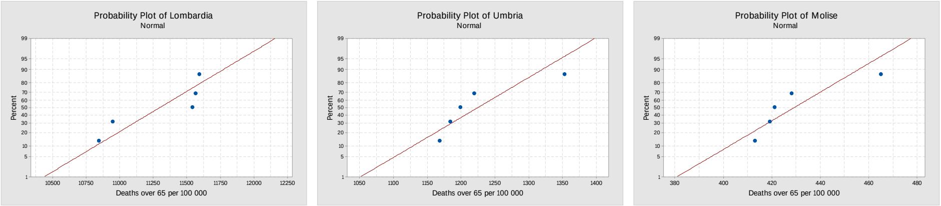

Figure 2: Normality check of data samples of deaths among over 65 for years 2015-2019

Normally distributed data

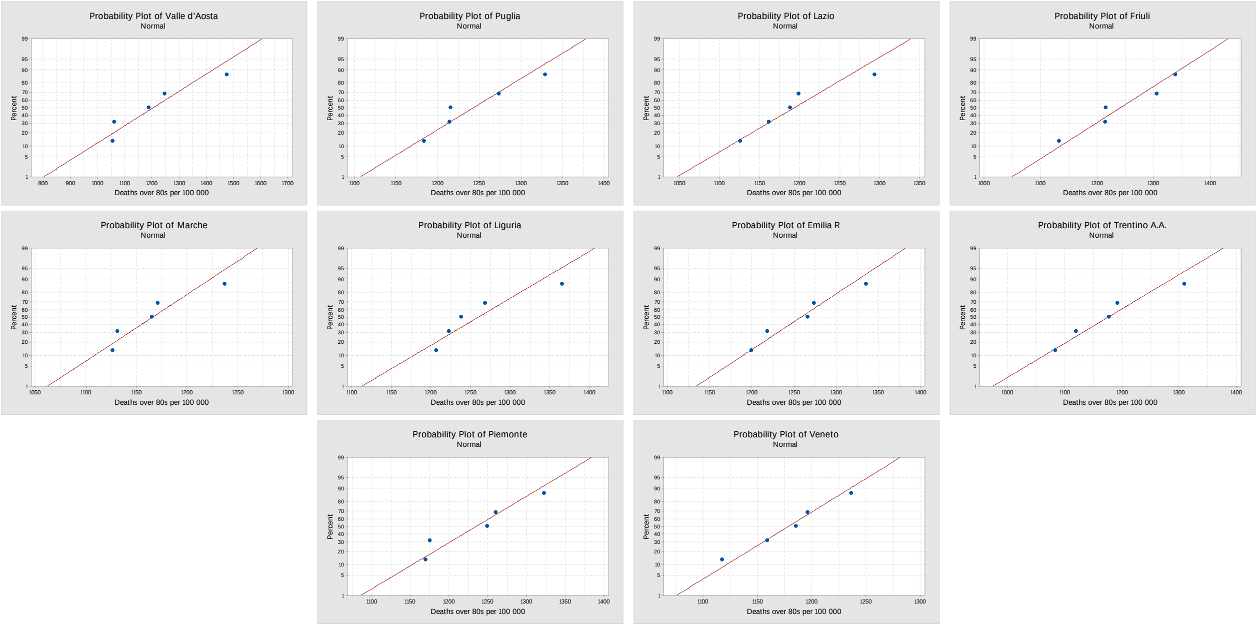

Not normally distributed data

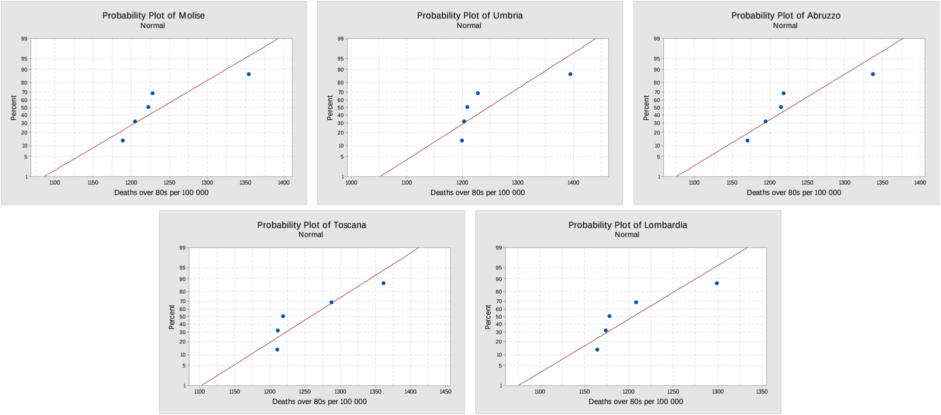

Figure 3:Normality check of data samples of deaths among over 80 for years 2015-2019

According to the normality check we applied:

- <u>One sample t-test</u>
    - Abruzzo, Toscana, Valle d’Aosta, Puglia, Lazio, Friuli V.G, Veneto, Marche, Liguria, Emilia R., Trentino A. A., Piemonte for over 65;
    - Valle d’Aosta, Puglia, Lazio, Friuli V.G, Veneto, Marche, Liguria, Emilia R., Trentino A. A., Piemonte for over 80.

- <u>Wilcoxon’s Rank test</u>
    - Lombardia, Molise, Umbria for over 65;
    - Molise, Umbria, Abruzzo, Toscana, Lombardia for over 80

The null hypothesis of the difference between mean value 2015-2019 and value 2020 is not satisfied for Umbria, Lazio and Molise for both over 65 and over 80.

In Appendix 2 the Minitab® outputs of One-Sample T test and Wilcoxon Signed Rank Test are reported

2) ***Normality test of dependent and independent variables for Pearson or Spearman coefficients***

Normally distributed data

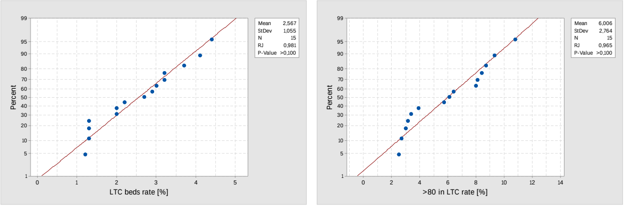

Not normally distributed data

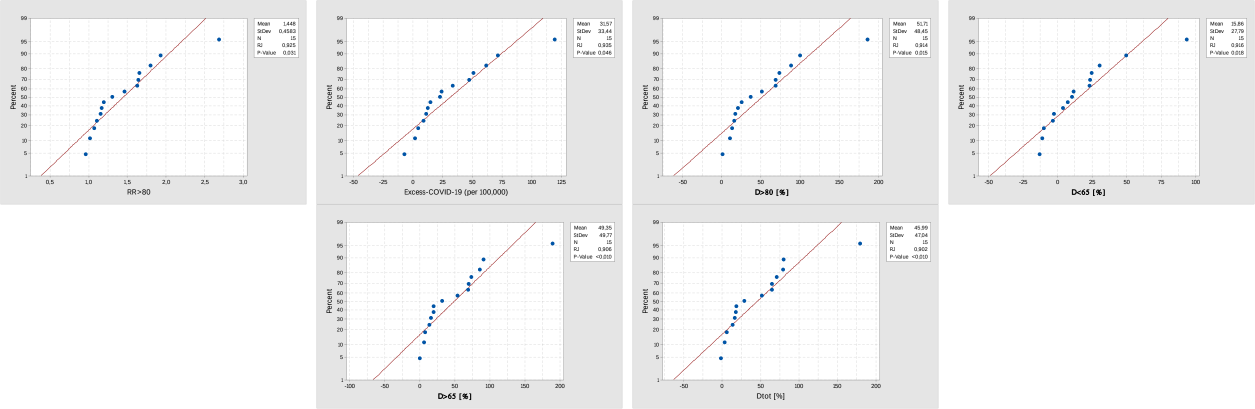

Figure 4: Normality check for dependent and independent variables

3) Power law vs Exponential fitting.

As example we report the comparison between power-law and exponential fitting of cumulative number of infections for Liguria and Lombardia. Together with the data fitting, fitting errors are also reported calculated as it follows:

$$Fitting\ Erros = \frac{(x_i - E_i)^2}{E_i}$$

Where x_i is the actual value and E_i the expected value. The plots clearly show that power-law fitting better reproduce the actual data. Similar results have been observed for all regions.

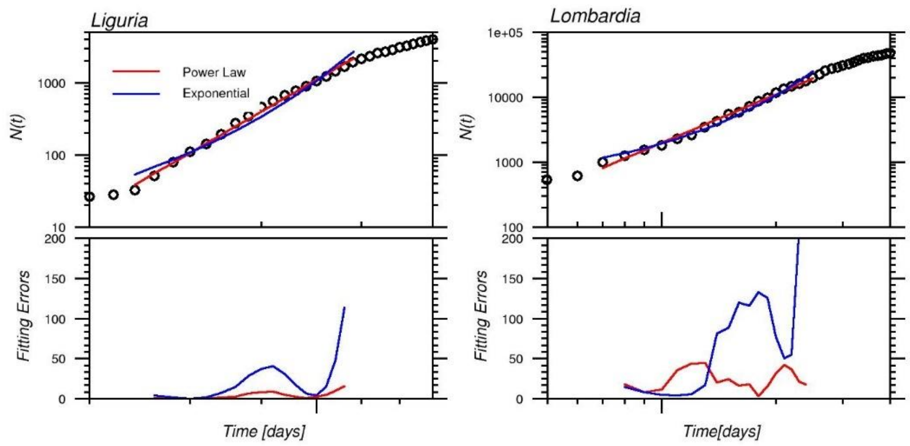

Figure 5: Comparison between power-law and exponential fitting of cumulative infections in Liguria and Lombardia. Data fitting and Fitting Errors are reported.

Appendix 2 – Minitab® Outputs for One-Sample T and Wilcoxon Signed Rank Test

Deaths among over 80

One-Sample T: Valle d'Aosta

```
Test of μ = 2307 vs ≠ 2307
Variable       N    Mean  StDev  SE Mean       95% CI            T      P
Valle d'Aosta  5  1203,6  172,1     77,0  (989,9; 1417,3)  -14,34  0,000
```

One-Sample T: Piemonte

```
Test of μ = 2035 vs ≠ 2035
Variable  N    Mean  StDev  SE Mean       95% CI            T      P
Piemonte  5  1235,0   63,9     28,6  (1155,6; 1314,4)  -27,98  0,000
```

One-Sample T: Trentino A.A.

```
Test of μ = 2109 vs ≠ 2109
Variable       N    Mean  StDev  SE Mean       95% CI            T      P
Trentino A.A.  5  1176,0   86,4     38,6  (1068,7; 1283,3)  -24,15  0,000
```

One-Sample T: Emilia R

```
Test of μ = 2047 vs ≠ 2047
Variable  N    Mean  StDev  SE Mean       95% CI            T      P
Emilia R  5  1258,2   53,1     23,8  (1192,2; 1324,2)  -33,19  0,000
```

One-Sample T: Liguria

```
Test of μ = 2060 vs ≠ 2060
Variable  N    Mean  StDev  SE Mean       95% CI            T      P
Liguria   5  1259,8   63,1     28,2  (1181,4; 1338,2)  -28,35  0,000
```

One-Sample T: Marche

```
Test of μ = 1697 vs ≠ 1697
Variable  N    Mean  StDev  SE Mean       95% CI            T      P
Marche    5  1166,0   44,4     19,9  (1110,8; 1221,2)  -26,73  0,000
```

One-Sample T: Veneto

```
Test of μ = 1530 vs ≠ 1530
Variable  N    Mean  StDev  SE Mean       95% CI            T      P
Veneto    5  1178,6   44,2     19,8  (1123,7; 1233,5)  -17,77  0,000
```

One-Sample T: Friuli

```
Test of μ = 1476 vs ≠ 1476
Variable  N    Mean  StDev  SE Mean       95% CI           T      P
Friuli    5  1240,8   81,8     36,6  (1139,2; 1342,4)  -6,43  0,003
```

One-Sample T: Lazio

```
Test of μ = 1141 vs ≠ 1141
Variable  N    Mean  StDev  SE Mean       95% CI          T      P
Lazio     5  1193,0   62,6     28,0  (1115,2; 1270,8)  1,86  0,137
```

One-Sample T: Puglia

```
Test of μ = 1372 vs ≠ 1372
Variable  N    Mean  StDev  SE Mean       95% CI           T      P
Puglia    5  1242,8   58,1     26,0  (1170,6; 1315,0)  -4,97  0,008
```

Wilcoxon Signed Rank Test: Molise

```
Test of median = 1382 versus median ≠ 1382
          N for   Wilcoxon         Estimated
        N   Test  Statistic      P     Median
Molise  5      5        0,0  0,059       1222
```

Wilcoxon Signed Rank Test: Umbria

Test of median = 1327 versus median ≠ 1327

	N	N for Test	Wilcoxon Statistic	P	Estimated Median
Umbria	5	5	1,0	0,106	1216

Wilcoxon Signed Rank Test: Abruzzo

Test of median = 1407 versus median ≠ 1407

	N	N for Test	Wilcoxon Statistic	P	Estimated Median
Abruzzo	5	5	0,0	0,029	1215

Wilcoxon Signed Rank Test: Lombardia

Test of median = 3231 versus median ≠ 3231

	N	N for Test	Wilcoxon Statistic	P	Estimated Median
Lombardia	5	5	0,0	0,000	1191

Deaths among over 65

One-Sample T: Valle d'Aosta

Test of μ = 1098 vs ≠ 1098

Variable	N	Mean	StDev	SE Mean	95% CI	T	P
Valle d'Aosta	5	576,3	72,4	32,4	(486,4; 666,2)	-16,11	0,000

One-Sample T: Piemonte

Test of μ = 942 vs ≠ 942

Variable	N	Mean	StDev	SE Mean	95% CI	T	P
Piemonte	5	558,12	17,65	7,89	(536,20; 580,03)	-48,64	0,000

One-Sample T: Trentino A.A.

Test of μ = 932 vs ≠ 932

Variable	N	Mean	StDev	SE Mean	95% CI	T	P
Trentino A.A.	5	503,0	34,0	15,2	(460,7; 545,2)	-28,21	0,000

One-Sample T: Emilia R

Test of μ = 970 vs ≠ 970

Variable	N	Mean	StDev	SE Mean	95% CI	T	P
Emilia R	5	561,06	20,13	9,00	(536,06; 586,06)	-45,42	0,000

One-Sample T: Liguria

Test of μ = 1004 vs ≠ 1004

Variable	N	Mean	StDev	SE Mean	95% CI	T	P
Liguria	5	592,0	23,6	10,6	(562,7; 621,4)	-39,00	0,000

One-Sample T: Marche

Test of μ = 837 vs ≠ 837

Variable	N	Mean	StDev	SE Mean	95% CI	T	P
Marche	5	545,80	16,45	7,36	(525,38; 566,22)	-39,59	0,000

One-Sample T: Veneto

Test of μ = 679 vs ≠ 679

Variable	N	Mean	StDev	SE Mean	95% CI	T	P
Veneto	5	515,07	9,60	4,29	(503,15; 526,99)	-38,18	0,000

One-Sample T: Toscana

Test of μ = 664 vs ≠ 664

Variable	N	Mean	StDev	SE Mean	95% CI	T	P
Toscana	5	558,21	20,57	9,20	(532,67; 583,74)	-11,50	0,000

One-Sample T: Friuli

Test of μ = 663 vs ≠ 663

Variable	N	Mean	StDev	SE Mean	95% CI	T	P
Friuli	5	554,8	33,3	14,9	(513,5; 596,2)	-7,26	0,002

One-Sample T: Abruzzo

Test of μ = 662 vs ≠ 662

Variable	N	Mean	StDev	SE Mean	95% CI	T	P
Abruzzo	5	573,47	16,83	7,53	(552,57; 594,37)	-11,76	0,000

One-Sample T: Lazio

Test of μ = 527 vs ≠ 527

Variable	N	Mean	StDev	SE Mean	95% CI	T	P
Lazio	5	530,84	13,89	6,21	(513,59; 548,09)	0,62	0,570

One-Sample T: Puglia

Test of μ = 601 vs ≠ 601

Variable	N	Mean	StDev	SE Mean	95% CI	T	P
Puglia	5	530,15	19,71	8,81	(505,67; 554,62)	-8,04	0,001

Wilcoxon Signed Rank Test: Lombardia

Test of median = 1447 versus median ≠ 1447

	N	N for Test	Wilcoxon Statistic	P	Estimated Median
Lombardia	5	5	0,0	0,000	498,6

Wilcoxon Signed Rank Test: Umbria

Test of median = 614,0 versus median ≠ 614,0

	N	N for Test	Wilcoxon Statistic	P	Estimated Median
Umbria	5	5	1,0	0,106	563,3

Wilcoxon Signed Rank Test: Molise

Test of median = 638,0 versus median ≠ 638,0

	N	N for Test	Wilcoxon Statistic	P	Estimated Median
Molise	5	5	1,0	0,106	596,6

KPSS Test performed by Gretl (Gnu Regression, Econometrics and Time-series Library) ver. 2017d

<u>*Deaths among over 80*</u>

```
Valle d'Aosta
Test KPSS for v1
T = 5
Lag Truncation  = 1
Test Statitics = 0,261679
                  10%      5%      1%
Critical Values : 0,380   0,464   0,579
P-value > .10

Lombardia
Test KPSS for v2
T = 5
Lag Truncation = 1
Test Statitics = 0,304338
                  10%      5%      1%
Critical Values : 0,380   0,464   0,579
P-value > .10

Piemonte
Test KPSS for v3
T = 5
Lag of truncation = 1
Test Statistics = 0,275734
                  10%      5%      1%
Critical Values: 0,380   0,464   0,579
P-value > .10

Trentino A.A.
Test KPSS for v4
T = 5
Lag of truncation = 1
Test Statistics = 0,285325
                  10%      5%      1%
Critical Values: 0,380   0,464   0,579
P-value > .10

Emilia R
Test KPSS for v5
T = 5
Lag of truncation = 1
Test Statistics = 0,298688
                  10%      5%      1%
Critical Values: 0,380   0,464   0,579
P-value > .10
```

Liguria

```
Test KPSS for v6
T = 5
Lag of truncation = 1
Test Statistics = 0,279702
                  10%      5%      1%
Critical Values: 0,380   0,464   0,579
P-value > .10
```

Marche

```
Test KPSS for v7
T = 5
Lag of truncation = 1
Test Statistics = 0,284154
                  10%      5%      1%
Critical Values: 0,380   0,464   0,579
P-value > .10
```

Veneto

```
Test KPSS for v8
T = 5
Lag of truncation = 1
Test Statistics = 0,24657
                  10%      5%      1%
Critical Values: 0,380   0,464   0,579
P-value > .10
```

Toscana

```
Test KPSS for v9
T = 5
Lag of truncation = 1
Test Statistics = 0,207578
                  10%      5%      1%
Critical Values: 0,380   0,464   0,579
P-value > .10
```

Friuli V.G.

```
Test KPSS for v10
T = 5
Lag of truncation = 1
Test Statistics = 0,211337
                  10%      5%      1%
Critical Values: 0,380   0,464   0,579
P-value > .10
```

Abruzzo

```
Test KPSS for v11
T = 5
Lag of truncation = 1
Test Statistics = 0,178284
                  10%      5%      1%
Critical Values: 0,380   0,464   0,579
P-value > .10
```

Umbria

```
Test KPSS for v12
T = 5
Lag of truncation = 1
Test Statistics = 0,176021
                  10%      5%      1%
Critical Values: 0,380   0,464   0,579
P-value > .10
```

Molise

```
Test KPSS for v13
T = 5
Lag of truncation = 1
Test Statistics = 0,152128
                  10%      5%      1%
Critical Values: 0,380   0,464   0,579
P-value > .10
```

Lazio

```
Test KPSS for v14
T = 5
Lag of truncation = 1
Test Statistics = 0,310493
                  10%      5%      1%
Critical Values: 0,380   0,464   0,579
P-value > .10
```

Puglia

```
Test KPSS for v15
T = 5
Lag of truncation = 1
Test Statistics = 0,193965
                  10%      5%      1%
Critical Values: 0,380   0,464   0,579
P-value > .10
```

Deaths among over 65

Valle d’Aosta

```
Test KPSS per v1
T = 5
Lag of truncation = 1
Test Statistics = 0,27041
                  10%      5%      1%
Critical Values: 0,380   0,464   0,579
P-value > .10
```

Lombardia

```
Test KPSS per v2
T = 5
Lag of truncation = 1
Test Statistics = 0,317794

                  10%      5%      1%
Critical Values: 0,380   0,464   0,579
P-value > .10
```

Piemonte

```
Test KPSS per v3
T = 5
Lag of truncation = 1
Test Statistics = 0,304118
                  10%      5%      1%
Critical Values: 0,380   0,464   0,579
P-value > .10
```

Trentino A. A.

```
Test KPSS per v4
T = 5
Lag of truncation = 1
Test Statistics = 0,303833

                  10%      5%      1%
Critical Values: 0,380   0,464   0,579
P-value > .10
```

Emilia R.

```
Test KPSS per v5
T = 5
Lag of truncation = 1
Test Statistics = 0,311559
                  10%      5%      1%
Critical Values: 0,380   0,464   0,579
P-value > .10
```

Liguria

```
Test KPSS per v6
```

```
T = 5
Lag of truncation = 1
Test Statistics = 0,30644
                  10%      5%      1%
Critical Values: 0,380   0,464   0,579
P-value > .10
```

Marche

```
Test KPSS per v7
T = 5
Lag of truncation = 1
Test Statistics = 0,299257
                  10%      5%      1%
Critical Values: 0,380   0,464   0,579
P-value > .10
```

Veneto

```
Test KPSS per v8
T = 5
Lag of truncation = 1
Test Statistics = 0,309034
                  10%      5%      1%
Critical Values: 0,380   0,464   0,579
P-value > .10
```

Toscana

```
Test KPSS per v9
T = 5
Lag of truncation = 1
Test Statistics = 0,277962
                  10%      5%      1%
Critical Values: 0,380   0,464   0,579
P-value > .10
```

Friuli V. G.

```
Test KPSS per v10
T = 5
Lag of truncation = 1
Test Statistics = 0,273769
                  10%      5%      1%
Critical Values: 0,380   0,464   0,579
P-value > .10
```

Abruzzo

```
Test KPSS per v11
T = 5
Lag of truncation = 1
Test Statistics = 0,288828
                  10%      5%      1%
Critical Values: 0,380   0,464   0,579
P-value > .10
```

Umbria

```
Test KPSS per v12
T = 5
Lag of truncation = 1
Test Statistics = 0,15311
                  10%      5%      1%
Critical Values: 0,380   0,464   0,579
P-value > .10
```

Molise

```
Test KPSS per v13
T = 5
Lag of truncation = 1
Test Statistics = 0,157066
                  10%      5%      1%
Critical Values: 0,380   0,464   0,579
P-value > .10
```

Lazio

```
Test KPSS per v14
T = 5
Lag of truncation = 1
Test Statistics = 0,115734
                  10%      5%      1%
Critical Values: 0,380   0,464   0,579
P-value > .10
```

Puglia

```
Test KPSS per v15
T = 5
Lag of truncation = 1
Test Statistics = 0,286167

                  10%      5%      1%
Critical Values: 0,380   0,464   0,579
P-value > .10
```
